# Three-Dimensional Quantification of Facial Morphology and Movements Using a Wearable Helmet

**DOI:** 10.1155/2022/2774713

**Published:** 2022-02-23

**Authors:** Marina Guihard, Jean-Michel Gracies, Marjolaine Baude

**Affiliations:** ^1^BIOTN Research Unit, UPEC University, 94000 Créteil, France; ^2^Rehabilitation Unit of Henri Mondor Hospitals, 1 Rue Gustave Eiffel, 94000 Créteil, France

## Abstract

This work proposes a 3D normative database of facial ranges of motion in adults free from facial disorders. Ten facial movements were analyzed, each targeting the activity of specific muscle groups innervated by the facial nerve. The experimental protocol included a test-retest reliability positioning procedure of 25 skin markers based on clinical expertise in facial morphology. Three maximal voluntary contractions were recorded for each facial movement studied, using a 3D facial motion capture helmet. We included 53 adults free from facial disorders (26 men; age 43 ± 14), evaluated twice one week apart. The reliability of marker positioning was expressed as absolute measurement errors. The range of motion vectors of all markers from the best rest to the maximal voluntary contraction was calculated for each muscle group. Primary, secondary, and tertiary markers were extracted for each facial movement. 3D Procruste and asymmetry indices were developed. This allowed the identification of common thresholds of 10% for the asymmetry index and of 6 mm for the Procruste index, beyond which facial motions would be considered abnormally asymmetric. The normative database quantifies facial motions and allows assessment of the degree of clinical disorders by comparison. This protocol is currently being investigated in patients with chronic unilateral peripheral facial paresis.

## 1. Introduction

Impairment of the facial nerve has functional, aesthetic, and emotional impacts which can lead to social isolation and depression [1]. Assessment of facial motions remains a challenge because it involves a high number of short muscle groups, often difficult to locate and with a small range of motion (ROM) [2, 3]. Besides, each face is unique in terms of dimensions, proportions, and even degree of symmetry. No standard model has been established.

Peripheral facial paresis (PFP) is a common disease that generally affects middle-aged patients, regardless of sex [4, 5]. In clinical practice, the degree of facial dysfunction is evaluated primarily by subjective clinical grading scales [6, 7]. Functional or plastic reconstructive surgical procedures attempt to bring solutions for acute local disorders and may produce significant improvements [8, 9] whereas physical rehabilitation is aimed at improving the neuromuscular control of facial muscles. Rehabilitation-induced motion recovery after facial paresis may then prove very slow, which requires precise quantification tools to detect motor skill improvements [10]. It is likely that the combination of validated quantification techniques and clinical scales should improve evaluation objectivity and reliability [11].

The objective tools are based on specific motion measurements in two dimensions [12, 13] and three dimensions [14–19], on shape analysis as for topography indices [20], using stereophotogrammetric procedures [21] or 3D mesh processing [22]. It is now established that 2D analysis is not sufficient to measure facial motions as 2D measurements ignore anterior-posterior axis displacements [10]. In 1999, Frey et al. designed a 2D device equipped with 2 mirrors and a calibration grid to compute the third dimension [23]. This device was marketed and used in a major international study on 241 PFP patients reported by Tzou et al. in 2012 [18]. 3D capture motion principle is based on the tracking of markers glued to the skin with several cameras placed around the head. For a long time, 3D analysis was limited to research studies as experimental conditions were unrealistic for wider use in clinical practice [24]. Furthermore, head motion during recordings compromised the reliability of the data. It is essential to define a common frame independent of head motions. In recent works, Ben Mansour et al. [25] compared three anatomical references composed of two rigid structures with bonded markers fixed in the maxillary, the auricle, and a pericranial band and determined that the device fixed to the jawbone is the best reference. Vimercati et al. [15] as well as Feng et al. [16] proposed a rigid support fixed on the head with reflective points. Trotman et al. performed a Procruste rotation to fit individual frames onto a standardized template to counteract head motions [19].

With the rise of biometric facial recognition and the development of custom avatars in videogames, technological advances have brought about new tools. In this work, we used the VICON Cara device, an instrumented helmet able to track small markers in 3D, even with large head motions (Vicon Oxford, UK). Aside from being the most recent technology specifically designed for facial motion analysis, this device offers a new flexibility, suitable for wide clinical measurement campaigns.

Beyond the acquisition system tool, the objective to describe the facial nerve function in terms of facial motions depends on the experimental protocol. The first challenge is to select marker types and positions. Most previous studies used adhesive and reflective markers of different diameters (from 3 mm [15, 19] up to 9 mm [17]). Tzou et al. reported that low contrast and shadows of facial creases sometimes interfered with the tracking process [18]. These authors tested active markers and reported the difficulty of attaching them to the skin. In their perspectives, Vimercati et al. proposed to use a pen with reflective ink [15] in order to ease marker positioning, to allow the use of smaller markers, and to decrease preparation time. In this work, we drew 2 mm diameter dots using a standard makeup pen using various colours depending on skin pigmentation.

The marker locations are key for the recording of maximal ranges of motion. Facial motion analysis involves more than 20 muscle groups on each side of the face, which overlap in a small volume. There are no known perfectly still facial points [26] although specific anatomical points are commonly studied (nasion, mouth commissures, base of the nose wings, frontal) [10, 27]. Their location is usually determined to describe the facial soft tissue structure for surgical reconstruction purposes [28] or for discrimination purposes of facial motion [29]. Our objective differs here, as we focus on assessing potential changes in facial neuromuscular control in PFP patients. Facial markers must be located at the point that will move the most during maximal muscle fiber recruitment in target muscle groups. Bandini et al. developed recent automatic detection marker algorithms and demonstrated a bias correlated with the severity of facial impairment [30]. We opted for a precise positioning technique based on careful inspection by an experienced senior physician (MB). We developed a precise positioning technique based on careful inspection by an experienced senior physician (MB). Twenty-five dots were traced on the face, of which 18 served for motion investigations. Rest and maximal voluntary contractions were recorded to estimate the ranges of motion for 10 facial muscle groups.

Symmetrization is deemed to be related to aesthetic appeal [22]. Therefore, many studies have been based on superimposing the two hemifaces and analyzing shapes using image processing [21, 31, 32] or computing the average distance of pairs of markers and the percentage of coincidence [33, 34]. The Procruste analysis duplicated the original landmarks in a mirror version reflecting along the sagittal plane [35] or the plane of maximum symmetry [36, 37] or an arbitrary plane outside of the face [21, 38]. This mirror technique does not fit our objective of assessing paretic facial motions, as the unaffected hemiface is influenced by the movements of the affected hemiface in both intended and unintended movements [19, 39]. Hyperactive reactions due to synkinesia or opposite directions of facial movements of the flaccid hemiface affect symmetry [40]. In this work, we propose to use a classical asymmetry index and a new Procruste index.

To the best of our knowledge, this work is the first attempt to quantify 3D motion for the main facial muscle groups. It provides a normative database with which facial disorders can be compared. It is part of the preclinical phase of the government-funded VISAGE project (NCT04074018).

## 2. Materials and Methods

### 2.1. Experimental Protocol

To assess facial movements, we identified 10 main muscle groups and defined the corresponding bilateral tasks. Subjects were to perform each task with a maximal voluntary contraction. Three maximal voluntary contractions were recorded lasting three seconds each, separated by five seconds of rest periods [10, 15].  Table 1 displays the target muscle groups and the description of the tasks in the order prescribed by the clinician.

**Figure 1:**
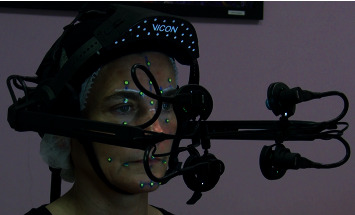
VICON Cara helmet device.

The Cara device, initially designed for film and game studios, is an instrumented 4 camera-helmet, weighing 1.2 kg, with comfortable fastening straps and a controllable rig of four on-board lights to adjust marker contrasts according to skin pigmentation (see Figure 1).

Fifty-three adults without PFP (26 men; age 43 ± 14) with no cervico-facial injuries or neurologic disorders participated twice at least one week apart (*n* = 53 participants, *n*′ = 106 evaluations) (ID-RCB 2018-A01815-50). An experienced rater manually drew 25 dots on the face using a makeup pencil. A specific procedure was followed to identify the best location of each marker based on the maximum recruitment of the target muscle fibers and of the facial morphology.  Table 2 displays the marker names and descriptions in relation to the photograph.

Subjects were first trained to perform the 10 tasks in front of a mirror for visual feedback. They were then seated comfortably on a chair, and the instrumented helmet was set upon their head. The helmet was mounted with a rigid connection to the head. The room light intensity was adjusted to achieve clear views from all cameras, avoiding subject discomfort.

### 2.2. VICON Cara Softwares

The Vicon Cara system comprises two softwares: Caralive records live captures with four backlighted cameras, and Carapost tracks all markers and provides their coordinates in a helmet-dependent orthonormal reference frame. A configuration step consisted of detecting the markers (size, brightness threshold) and checking their tracking along the capture.

The acquisition frequency was 50 Hz. A 2D anatomical reference plane was defined using 3 points: 2 external end points of the eyes and N0. The origin of the coordinate system was the centroid of the three points. The *X*-axis was the line between the 2 external canthi. The *Y*-axis was the line perpendicular to the *X*-axis that crossed the origin of the coordinate system in the reference plane. The *Z*-axis was the line perpendicular to the reference plane that crossed the origin of the coordinate system. All coordinates were converted to a text file format.

### 2.3. Data Processing

**Figure 2:**
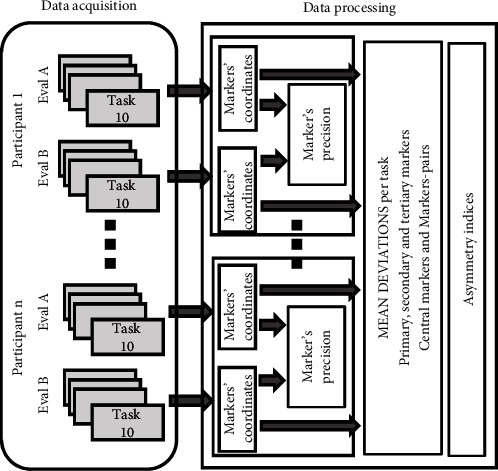
Data processing architecture.

Specific software was developed using MATLAB. The development of automation modules at different stages of motion processing is described in Figure 2.

As motion was recorded in mobile reference frames, the first step was to define a common reference frame from one evaluation to another for the same participant. A change in the initial frame proposed by the Carapost software was performed. The origin was placed on the N0 marker, which is the most central and easiest to position [10, 13, 27]. The *X*-axis remained unchanged (line from N0 to the external canthus), and the *Y*-axis was positioned along the line N0-F0 to define the sagittal plane as (N0, $\overset{⟶}{y}$, $\overset{⟶}{z}$) plane.

The second step consisted of locating the maximal movement on the three contractions according to task-dependent clinical reference markers and optimal rest. In fact, it appeared that after a maximal contraction, markers rarely returned to their initial positions, making complete relaxation of the group of muscles difficult to achieve. Some recordings showed optimal rest at the beginning, others between contractions. For each recording, an automation procedure was developed to compute the mean of the maximal motion and of the optimal rest over a stable period. Visual as well as automatic verifications were performed to detect outliers. A vector of motion per marker was then computed from the rest position to the maximum motion position for each task as in (1) where Mark_Rest_*i*__ and Mark_Cont_*i*__represent the coordinates of marker *i*, respectively, at rest and at the maximum of contraction.

(1) $$\begin{array}{c} \vec{∆{\text{Mark}}_{i}}=\vec{{\text{Mark}}_{{\text{Cont}}_{i}}{\text{Mark}}_{{\text{Rest}}_{i}}}. \end{array}$$

This process was applied to the 10 tasks of the 106 evaluations to get the mean and standard deviation of all of the 3D spherical coordinates. From the obtained module range of values, relevant markers were determined and classified in three levels as in (2) where Mark_prim_^*t*^, Mark_second_^*t*^, and Mark_tert_^*t*^ represent the set of, respectively, primary, secondary, and tertiary markers for a given task *t*.

(2) $$\begin{array}{c} {\text{Mark}}_{i}^{t}\in {\text{Mark}}_{\text{prim}}^{t}\Leftrightarrow \frac{3}{4}\;M\_\max^{t}\leq \left\|\vec{∆{\text{Mark}}_{i}^{t}}\right\|,\\ {\text{Mark}}_{i}^{t}\in {\text{Mark}}_{\text{second}}^{t}\Leftrightarrow \frac{1}{2}M\_\max^{t}\leq \left\|\vec{∆{\text{Mark}}_{i}^{t}}\right\|<\frac{3}{4}\;M\_\max^{t},\\ {\text{Mark}}_{i}^{t}\in {\text{Mark}}_{\text{tert}}^{t}\Leftrightarrow \frac{1}{4}M\_\max^{t}\leq \left\|\vec{∆{\text{Mark}}_{i}^{t}}\right\|<\frac{1}{2}\;M\_\max^{t}\\ with\;M\_\max^{t}=\underset{i=1\;\text{to }24}{\max}\left\|\vec{∆{\text{Mark}}_{i}^{t}}\right\|. \end{array}$$

The last step was to quantify facial asymmetry. Marker pairs were identified from the previous analysis that define the degree of primary, secondary, and tertiary symmetry in relation to their level for each task. Two asymmetry indices were calculated, one for the rest position (3) and one for the maximal voluntary contraction position (4) where *C* represents the central marker associated with the pairs of markers, *R* the dominant/healthy side, and *NR* the other side.

(3) $$\begin{array}{c} \text{IdAsy}{\text{m}}_{\text{Rest}}=\left|\frac{\left\|\vec{{\text{Mark}}_{C}{\text{Mark}}_{R}}\right\|-\left\|\vec{{\text{Mark}}_{C}{\text{Mark}}_{NR}}\right\|}{\left\|\vec{{\text{Mark}}_{C}{\text{Mark}}_{R}}\right\|+\left\|\vec{{\text{Mark}}_{C}{\text{Mark}}_{NR}}\right\|}\right|*100\;\left(\%\right), \end{array}$$

(4) $$\begin{array}{c} \text{IdAsy}{\text{m}}_{\text{Cont}}=\left|\frac{\left\|\vec{∆{\text{Mark}}_{R}}\right\|-\left\|\vec{∆{\text{Mark}}_{NR}}\right\|}{\left\|\vec{∆{\text{Mark}}_{R}}\right\|+\left\|\vec{∆{\text{Mark}}_{NR}}\right\|}\right|*100\;\left(\%\right). \end{array}$$

These indices were calculated for all evaluations. The mean and standard deviation provided a range of values for a population of patients without PFP, from which asymmetry indices of PFP patients can be later compared. However, these indices may not be adequate to evaluate the progress of facial motion recovery. In fact, unilateral peripheral facial paresis can cause a deformity in which central markers no longer lie on the *Y*-axis, and the movement of marker pairs on the paretic side becomes inverted compared to expected normal movements. Some patients have flaccid muscles, whereas others have hyperactive reactions characterized by synkinesia. Then, another important objective was to develop indices that can quantify facial asymmetries in patients with PFP. We proposed a new asymmetry index IdPro, based on a Procruste analysis [41] with the central, primary, secondary, and tertiary markers of each task. The computed distance between the dominant and the nondominant sides gave a quantified value reflecting the degree of similarity between the 2 volumes.

## 3. Results

### 3.1. Marker Positioning Accuracy

Table 3 shows the differences in the marker positioning test-retest module between the evaluations performed one week apart for each marker. Marker diameters are estimated at 2 mm. Two markers have been removed for the mean computation (0.16%).

### 3.2. Marker Range of Motion

**Figure 3:**
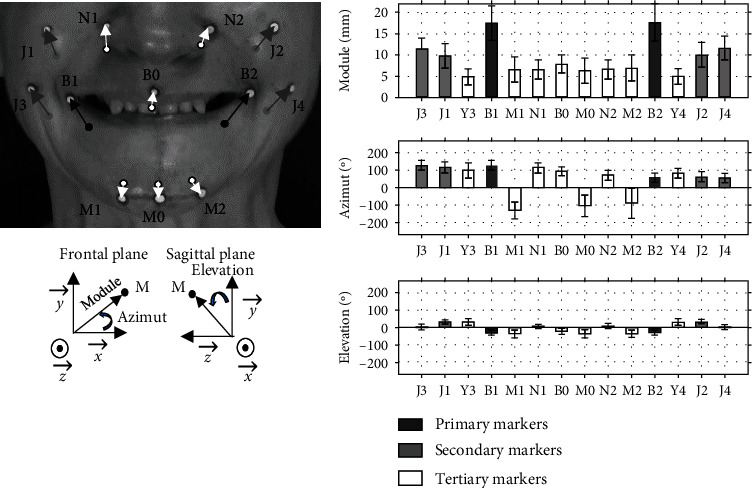
Analysis of the zygomaticus motion. The bar graphs represent the mean ± standard deviation of the free PFP participants for the primary, secondary, and tertiary markers determined as in (2) in spherical coordinates, *n*′ = 106.

For each task, a posteriori video analysis was performed to detect motions that were not well performed. 0.6% of the tasks (6 tasks out of 1006) were deleted because the participant was unable to follow the instructions (facial apraxia). It mainly related to the depressor anguli oris task, which is not a common movement in everyday life. Physiological cocontractions of other facial muscles were observed during the requested tasks, leading to the displacement of nonprimary markers. The location and the intensity of cocontractions varied depending on the task, the most obvious being during corrugator activation with the lower face tensed up (3% marker deleted) and for the depressor anguli oris task with cocontraction of the corrugator (6% marker deleted). The rise in the eyebrow—corresponding to the frontalis—was the best performed task (0.05% marker removed).  Figure 3 represents the results for the zygomaticus task. It shows the displacement of the lower face markers in the chosen reference frame.

Tables 4–6 display the database built for each task according to the part of the face involved (upper, middle, and lower part). Mean and SD are given for the 106 evaluations to take into account the intrasubject variation of motion from one evaluation to the other, averaging 7% for all the tasks.

### 3.3. Asymmetry Indices

**Figure 4:**
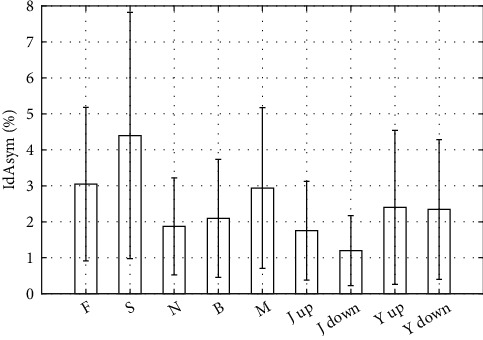
Asymmetry indices at rest for each pair of markers (see Table 2). Data presented as mean ± standard deviation (%), *n*′ = 106.

**Figure 5:**
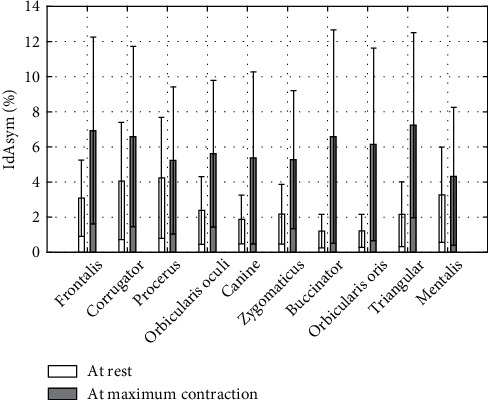
Asymmetry at rest and maximal contraction per task for pairs of markers of interest (see Table 1). Data presented as mean ± standard deviation (%), *n*′ = 106.

**Figure 6:**
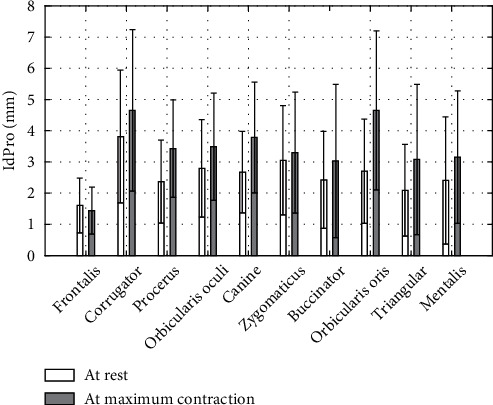
Procruste indices per task. Data presented as mean ± standard deviation (mm). Volumes of central and primary/secondary/tertiary side markers reported in Tables 4–6, *n*′ = 106.

**Figure 7:**
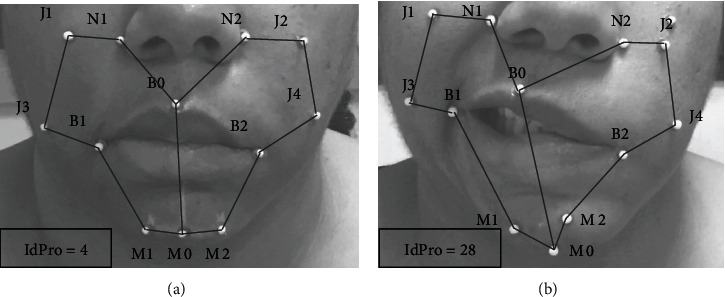
Example of Procruste indices (mm) in a patient with left chronic PFP: (a) rest; (b) maximal contraction of the zygomaticus.

Figure 4 represents the asymmetry indices computed at rest from Equations (3) to (4) for all the paired markers. Central markers associated to the marker-pairs F, S, B, and M are, respectively, F2, S0, B0, M0, and N0 for all the others.  Figure 5 reports the asymmetry indices computed from Equations (3) to (4) at rest and maximal motion conditions for the markers for interest of each task (see Table 1). In Figure 6, Procruste indices at rest and at maximal contraction are displayed for each task. Primary, secondary, and tertiary lateral and central markers were collected, and the Procruste distance was calculated to match the set of markers of both the nondominant and dominant sides. The Procruste index at rest taking into account all the markers of each side of the face is 2.47 ± 1.16 mm.  Figure 7 illustrates an example of the Procruste distance for a patient with chronic left peripheral facial paresis during the zygomaticus task.

## 4. Discussion

### 4.1. ROM Analysis

This work provides a database of 10 muscle displacements from rest to maximal voluntary contraction in terms of ROM and orientation. The ROMs of all pairs of markers for each task (see Tables 4–6) are consistent with clinical observations [11]. The standard deviations are related to the diversity of motion performance between the participants.  Figure 3 gives an example of how these data will be used in the clinic. It shows which markers are involved and at what level according to Equations (2) for the zygomaticus motion in a free of PF population.

Central markers are involved in multiple ways. M0 and B0 are the most mobile for lower face tasks. For the middle face, procerus and canine tasks involve the displacement of the greatest number of markers. These tasks reflect global facial mobility. Of note, the upper face tasks involve significant cocontractions of the lower face muscles whereas lower face tasks are performed without much cocontractions of the upper face muscles. This suggests that it is “easier” to perform lower face tasks as it is the most mobile and active part of the face: a maximum of 17.6 mm ROM for markers B in the zygomaticus task and 8.6 mm for markers F in the frontalis task. Inflating and sucking the chest involve all the lower facial muscles. These tasks involve the largest 3D displacements. Cheek sucking (buccinator) is difficult to perform without pursing the lips, leading mentalis markers (M) to become primary markers for this task, unlike the inflating task (orbicularis oris).

Few numerical data from the literature are truly comparable. Some show data in the frontal plane only [13, 15, 17] or for specific vector characteristics [14] or separately for each participant [17, 27] or results after scaling each face to an average facial size [19]. Coulson et al. present ROM results of main markers for zygomaticus, frontalis, canine, and OP tasks [10]. Their order of magnitude for the mean is overall lower than our results (from 2 mm for the canine task up to 4 mm for the zygomaticus task). This may be due to the various types and sizes of markers (7 mm versus 2 mm) and the accuracy of positioning. In any case, the standard deviations of all comparable data are similar (from 2.5 to 4.2 mm) for a similar number of participants (42 versus 53). The same observations can be made with the study by Sforza et al., from their reported ROM for the zygomaticus and OP tasks [42]. To some extent, the device first designed by Frey et al. gives similar results with the computation of the third dimension from two 2D analysis [43].

The present work goes further than previous studies, as it presents the mobility of the face through a 3D analysis of 10 groups of muscles. Beyond the markers of interest, all the associated facial motions have been investigated here.

### 4.2. Symmetry Analysis

The symmetry of the face was quantified through asymmetry indices as performed in clinical grading scales [6]. Figure 4 shows the pairs of marker asymmetry at rest. The face at rest is generally symmetric, and the standard deviations reflect natural differences between the participants. The highest values were found for pairs of markers for which N0 is not the central reference (F: 3 ± 2.1%, S: 4.4 ± 3.4%, M: 2.9 ± 2.3%).

For contraction tasks, the indices presented the asymmetry for pairs of markers of interest in Figure 5. Asymmetry indices IdAsym at maximal contraction are twice greater than at rest (5.9 ± 0.8% versus 2.6 ± 0.9%). This reflects the natural asymmetry of human facial contractions. Differences between rest and contraction asymmetry indices range from 1% for procerus and mentalis tasks to 5% for triangular, buccinator, and orbicularis oris tasks. Cheek movements involve a high 3D mobility of soft tissues, and the depressor anguli oris task is the most difficult task to perform. SD are representative of the population heterogeneity. Our definition of asymmetry indices was similar to that proposed by Sforza et al. [42] although these authors put forward a mean value for a group of markers for each task. For the zygomaticus task, its mean value was 5.57%, similar to our results (5.12%).

In view of these results, the IdAsym threshold of 10% seems an indicator of abnormal facial symmetry. Thus, these indices indicate objective values on local asymmetry, to be compared with clinical grading scales.

Traditional mirror techniques are inappropriate for our final purpose; as for paretic patients, the unaffected hemiface is influenced by the movements of the affected hemiface in both intended and unintentional movements [19, 39]. Hyperactive reactions due to facial synkinesia or facial spasms [40], as well as flaccid area behavior, require the definition of adapted asymmetry indices. We propose a Procruste index computed for each task. This method is well known in geometric shape analysis [41] and has been developed for the assessment of bilateral symmetry [44] by comparing the original configuration with a mirror reflection configuration. Previous works proposed an asymmetry index focused on the lower face [36, 45] or on the whole face [19]. We propose a Procruste index IdPro without any projection of one side to another but comparing left and right 3D specific shapes including central markers. Figure 6 provides data for each task taking into account all primary, secondary, and tertiary markers of each side. All mean values are comprised between 1.61 and 4.65 mm. The difference between rest and maximum contraction of this index is twice less that of the previous local index (26% versus 56%) with similar SD. IdPro index is more representative of facial asymmetry as it compares volumes, whereas IdAsym index compares vector modules. In view of these results, the IdPro threshold of 6 mm seems an indicator of abnormal facial symmetry. Facial synkinesia can also be quantified through an analogous Procruste study as proposed on clinical grading scales [6].

Finally, Figure 7 illustrates the relevance of this index in defining a pathological smile. The IdPro index at rest (4 mm) is in agreement with the mean value of Figure 6 (3.0 ± 1.8 mm), and in contraction, this index is 8.7 times higher (28 mm versus 3.3 ± 2.0 mm). This is in agreement with the literature showing greater asymmetry in maximal contraction than at rest for patients with facial paresis [35].

### 4.3. Protocol Assessment

The objective to measure the facial motions resulted in the definition of 10 tasks, each of them focusing on a facial muscle activation reflecting facial nerve function. In the literature, the number of markers varies from a few dots distributed on anatomical points [15, 17, 26, 27] to high number of dots (nd = 64 [35], nd = 109 [46]). We chose 25 markers (drawn dots)—seven central and 18 lateral—to cover all the areas observed in the clinic without excessively complicating the practical experimental protocol. The size, type, and location of the markers were drawn from over 10 years of clinical experience for a senior facial paresis clinical expert (MB). The location of the lateral markers was derived from the clinical observation of the most mobile areas and from the morphology of the face. Drawing the facial dots directly on the skin reduced preparation time and probably provided better accuracy than 3D hemispherical or spherical markers and minimized the tracking error, although this comparison was not formally performed.

The VICON Cara helmet was developed for facial motion tracking toward the design of video games. To our knowledge, no medical application had been developed to date. This tool enables multiple assessments with the same environment, and its wearability brings new flexibility to enable wide clinical measurement campaigns. The reference frame is on the helmet, free from head motion. Furthermore, the exact positioning of the helmet is not a problem, as we aim to quantify ROM from one evaluation to the next.

In the literature, performed tasks have been selected to demonstrate the precision of the measurement tool [15] or to focus on specific movements, primarily to assess the effects of surgical procedures. The most studied facial expressions have been smiling, raising eyebrows, and closing the eyes [12, 14, 35]. To our knowledge, no studies have investigated buccinator and orbicularis oris muscle groups, involved in cheek and mouth functions. Mentalis and depressor anguli oris are also relatively unexplored. This protocol is therefore more complete than most the prior literature in the field.

A scaling to the average facial size has occasionally been proposed in the literature to eliminate the effect of differences in facial dimensions, using either a mean ratio between facial height and width [42] or an averaged Procruste distance of the face [19]. We did not adopt such a scaling, as muscle contraction-induced motions correlate not only with muscle dimensions but also with muscle activation [47]. No a priori relations between face dimensions and muscle ROM can be set, and scaling may add processing errors. We elected to handle facial morphological differences by working on a sufficient number of subjects to cover a large population of middle-aged adults. The standard deviations that were derived here appear to be greater than with an *a priori* scaling but may be more representative of the variety of facial muscle ROM.

The convenience sample of free from PFP subjects was selected to be balanced between men and women (26 men, *n* = 53) and middle-aged (43 ± 14) to be comparable with the PFP population. Previous studies have shown that age has an impact on facial dimensions and that male has greater facial motions than female [42, 43].

Another challenge was the definition of relevant indices capable of quantifying PFP in the clinical setting. Some authors proposed specific distances, angles, or ROM as a function of a specific facial area: mouth [15] and lower or upper face [14, 18]. Others proposed the marker excursion of all markers studied [10, 43]. Symmetry was mostly quantified following the mirror principle from one side to another [33, 34]. Our results determine the main markers involved for each movement, sorted according to their degree of motion (see Tables 4–6). The module and the arguments give the magnitude and orientation of each vector (see Figure 3). This direction of movement is essential as for PFP patients; the unaffected hemiface may be influenced by the movements of the affected hemiface in both intended and unintentional movements [19, 39]. Regarding asymmetry indices, our contribution proposes a Procruste study related to each task. Finally, we propose two reference thresholds as indicators of abnormal facial asymmetry.

## 5. Limitations and Strengths of the Study

### 5.1. Protocol Safety and Feasibility

The first concern was to evaluate how consistent the assessment protocol would be with evaluation of PFP patients in the clinical setting. The total time required for the clinic visit is estimated to be 45 minutes, including 15 minutes of marker setting and 20 minutes of motion recordings. The helmet was well accepted by our healthy participants (1.2 kg). Only one of them, with a history of chronic headache, reported light headache due to light intensity, which was adjusted accordingly during the retest evaluation.

The second concern was about the accuracy of marker positioning. Table 3 displays the mean of the 3D error modules between evaluations A and B. The mean total error is 2.65 ± 0.60 mm. Four key factors may be involved: marker shape and size (Ø 2 mm), reference frame readjustment, evaluator thoroughness, and skin properties. The first two are intrinsic to the process, the third depends on the degree of experience of the evaluator, and the last one on the facial features of the subject. The same order of magnitude was observed for marker pairs in a previous study [15]. Positioning errors from one evaluation to another are greater for markers located on soft tissues (B, M) or far from anatomical landmarks (J) than for markers easy to ascertain (F, S, N, Y). Vimercati et al. report the same conclusions for markers located at the apex of the cheek bone and at the mandibular joint [15]. In the literature, errors have been expressed through a statistical intraclass correlation [15], by identifying specific distances and angles [14, 16] or by the percentage of position error of each marker in the frontal plane [14]. These experimental protocols removed all markers and recorded motions in a time interval of a few minutes and without removing the rigid support on which the reference frame was based [14–16]. This seems impractical in the clinic as the patient often returns to the clinic several months after the previous visit. The process has to be reproducible from one examination to another. To our knowledge, no study reports 3D absolute positioning errors with the whole process replicated one week apart.

### 5.2. Limitations

The limits of our study relate to the facial complexity. The uniqueness of each face requires a specific positioning of the markers by a senior expert. Another limit is the time consumption of our data analysis, which was estimated around one hour in total, including the 40-minute clinical time with the patient. Our tested population should also be extended both in terms of number and of diversity (including Hispanic, Black, and Caucasian populations) to include ethnical differences [48].

### 5.3. Future Developments

Future work will complete this database, providing synkinesia indices and evaluating spontaneous facial movements by measuring contraction velocities [14, 19] and nonvoluntary emotion-induced movements. This protocol is currently being investigated for PFP patients (clinical phase of the government-funded VISAGE project, *n* = 82) with the objective of quantifying the effect of two rehabilitation programs on the recovery of facial motion in chronic stages. We will carry out a correlation study between the 3D findings and conventional clinical grading scales.

Finally, the emergence of 3D cameras will bring easier to implement devices in the next decade. Future comparisons could be made with our findings [49].

## 6. Conclusions

An objective and reliable tool allowing to quantify facial motions usable in the clinical setting is actually lacking. Previous 3D capture motion devices were difficult to utilize for both experimental and data processing time-consumption reasons. We used a wearable instrumented helmet specially designed for tracking facial motions and proposed an experimental protocol to assess 10 facial muscle groups with associated asymmetry indices. To our knowledge, this work provides the most complete database of nonparetic facial motions from rest to maximal voluntary contraction. Measurements of local asymmetry of pairs of markers and overall asymmetry based on volume Procruste indices complete this facial mobility study. By comparing PFP recordings to this normative database, the proposed protocol will allow quantifying the severity of PFP disorders and assessing therapeutic options such as rehabilitation programs, botulinum toxin injections, or facial surgeries.

## Figures and Tables

**Table 1:** Muscle and task correspondence.

Target muscle groups	Task description	Markers of interest
Frontalis	Eyebrow raising	F3, F4
Corrugator	Eyebrow frowning	S1, S2
Procerus	Nose crinkling	S1, S2
Orbicularis oculi	Eyes closure	Y1, Y2
Canine	Nose wings up (detect smell)	N1, N2
Zygomaticus	Smile mouth open	B1, B2
Buccinator	Cheek sucking	J1, J2
Orbicularis oris	Cheeks inflating	J1, J2
Triangular	Corners of the mouth lowering	B1, B2
Mentalis	Lower lip curling	M1, M2

**Table 2:** Marker positions.

**Lateral markers**			
F3	Forehead right side	F4	Forehead left side
S1	Eyebrow right side	S2	Eyebrow left side
N1	Nose right side	N2	Nose left side
B1	Right mouth corner	B2	Left mouth corner
M1	Chin right side	M2	Chin left side
J1	Cheek right side high	J2	Cheek left side high
J3	Cheek right side low	J4	Cheek left side low
Y1	Right eyelid	Y2	Left eyelid
Y3	Right eye	Y4	Left eye

**Central markers**		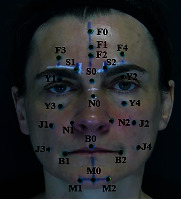	
F0	Forehead center high		
F1	Forehead middle center		
F2	Forehead center low		
S0	Eyebrow center		
N0	Nose center		
B0	Mouth center		
M0	Chin center		

**Table 3:** Marker positions.

Central markers		Rignt markers		Left markers	
F0	1.63 ± 1.48	F1	2.51 ± 3.16	F2	2.38 ± 3.26
S0	1.05 ± 0.47	F3	3.38 ± 1.39	F4	2.67 ± 1.24
B0	2.25 ± 1.19	S1	2.08 ± 1.10	S2	2.04 ± 1.36
M0	3.49 ± 1.83	N1	2.48 ± 1.12	N2	2.39 ± 1.13
		B1	2.94 ± 1.21	B2	3.20 ± 1.84
		M1	3.50 ± 1.73	M2	3.64 ± 1.80
		J1	3.30 ± 1.07	J2	2.79 ± 1.43
		J3	3.66 ± 1.83	J4	3.90 ± 2.10
		Y1	2.71 ± 1.22	Y2	2.31 ± 1.07
		Y3	2.25 ± 0.94	Y4	2.41 ± 1.18

Marking accuracy between the two evaluations one week apart, mean ± standard deviation of the modules (mm), *n* = 53.

**Table 4:** Upper face database.

**Frontalis**						**Corrugator**					
Primary markers		Secondary markers		Tertiary markers		Primary markers		Secondary markers		Tertiary markers	
F3	8.60 ±2.71	F2	6.15 ±2.53	F1	4.19 ±2.47	F3	9.73 ±2.16			Y3	3.45 ± 2.20
F4	8.73 ±2.65			S0	4.32 ±1.72	F4	9.68 ±2.61			Y4	3.43 ±2.28
S1	10.25 ±2.74			B0	3.53 ±1.11	S1	7.10 ±1.83			F2	3.38 ±1.84
S2	10.40 ±2.58			M0	4.72 ±1.58	S2	7.36 ±2.05				
Y1	9.22 ±2.54			B1	4.13 ±1.32	Y1	7.96 ±2.39				
Y2	9.45 ±2.59			B2	4.14 ±1.38	Y2	7.37 ±2.56				
				M1	4.68 ±1.58						
				M2	4.73 ±1.59						
				J3	4.01 ±1.39						
				J4	3.96 ±1.46						
											

**Orbicularis oculi**											
Primary markers		Secondary markers		Tertiary markers							
F3	9.45 ±2.66	S1	8.01 ±2.53	F1	3.98 ±2.21						
F4	9.22 ±3.01	S2	8.20 ±2.66	F2	5.03 ±2.35						
J1	9.39 ±2.99	J3	6.61 ±2.71	S0	3.47 ±1.76						
J2	8.98 ± 2.80	J4	6.06 ±2.32	B0	3.36 ± 2.30						
Y1	11.57 ±2.55			M0	3.52 ±1.94						
Y2	11.40 ±2.77			N1	5.67 ±2.52						
Y3	10.30 ±1.83			N2	5.21 ±2.47						
Y4	10.22 ±1.83			B1	4.65 ±3.18						
				B2	4.19 ±2.65						
				M1	3.44 ±1.85						
				M2	3.42 ±1.78						

Data presented as mean ± standard deviation of modules (mm), *n*′ = 106.

**Table 5:** Middle face database.

**Procerus**						**Canine**					
Primary markers		Secondary markers		Tertiary markers		Primary markers		Secondary markers		Tertiary markers	
F3	8.43 ±2.79	F1	5.46 ±2.47	F0	3.62 ±1.92	N1	10.01 ±2.31	F2	5.97 ±2.24	F0	3.45 ±1.59
F4	8.19 ±2.91	F2	6.66 ± 2.70	S0	4.81 ± 1.70	N2	9.74 ±2.26	B0	6.47 ±3.06	F1	4.94 ±1.96
S1	8.49 ±2.52	B0	6.28 ±3.31	M0	4.84 ±2.77	J1	10.24 ±3.36	F3	5.88 ±2.66	S0	4.34 ±1.41
S2	8.66 ±2.68	B1	7.30 ±3.65	M1	4.75 ±2.48	J2	9.60 ±3.01	F4	5.63 ±2.62	M0	4.75 ±3.03
N1	9.04 ±3.12	B2	7.50 ±3.41	M2	4.70 ±2.59			S1	6.90 ±2.19	M1	4.68 ±2.99
N2	9.05 ±3.05	J3	7.26 ±2.63					S2	7.05 ±2.43	M2	4.64 ±2.97
J1	10.14 ±3.12	J4	7,00 ±2.34					B1	7,00 ±3.47	Y1	5.12 ±2.39
J2	9.80 ±2.94	Y1	7.78 ±2.66					B2	6.99 ±3.46	Y2	5.19 ±2.53
		Y2	7.32 ±2.53					J3	7.01 ±3.41		
		Y3	6.23 ±2.12					J4	6.73 ±2.73		
		Y4	6.05 ±2.22					Y3	5.84 ± 2.70		
								Y4	5.47 ±2.27		

Data presented as mean ± standard deviation of modules (mm), *n*′ = 106.

**Table 6:** Lower face database.

**Buccinator**						**Orbicularis oris**					
Primary markers		Secondary markers		Tertiary markers		Primary markers		Secondary markers		Tertiary markers	
M0	16.53 ±6.99			B0	6.95 ±2.68	B1	15.05 ± 3.60	B0	9.80 ±3.78	M0	7.28 ±3.79
B1	12.24 ±2.88			J1	4.00 ±1.61	B2	15.19 ±3.45	J1	7.99 ±3.22	N1	3.83 ±2.02
B2	12.80 ±3.18			J2	4.31 ±1.91	J3	13.02 ±3.54	J2	7.85 ±2.97	N2	3.45 ±2.09
M1	15.42 ±6.47					J4	12.90 ±3.28			M1	6.98 ±3.35
M2	15.61 ±6.69									M2	6.96 ±3.55
J3	12.31 ±3.75										
J4	12.22 ±3.78										

**Zygomaticus**						**Triangular**					
Primary markers		Secondary markers		Tertiary markers		Primary markers		Secondary markers		Tertiary markers	
B1	17.53 ±4.17	J1	9.91 ±2.79	B0	7.89 ±2.11	B1	10.54 ±4.34	M0	7.18 ±3.84	B0	4.46 ±2.06
B2	17.64 ±4.39	J3	11.43 ±2.59	M0	6.35 ±2.99	B2	10.46 ±4.42	J3	5.95 ± 2.80		
		J2	10.15 ±2.91	N1	6.74 ±2.21			J4	5.90 ±2.59		
		J4	11.56 ±2.73	N2	6.67 ±2.26			M1	6.43 ±3.39		
				M1	6.62 ± 2.90			M2	6.39 ±3.38		
				M2	6.88 ±2.91						
				Y3	5.01 ±1.89						
				Y4	5.01 ±1.86						

**Mentalis**											
Primary markers		Secondary markers		Tertiary markers							
M0	12.05 ±3.64			B0	4.38 ± 2.20						
M1	10.86 ±3.49			B1	5.49 ±2.97						
M2	10.69 ± 3.60			B2	5.34 ±2.89						
				J3	3.62 ±1.85						
				J4	3.65 ±1.86						

Data presented as mean ± standard deviation of modules (mm), *n*′ = 106.

## Data Availability

The PC_VISAGE data used to support the findings of this study may be released upon application to the DRCI-URC AP-HP CHU Henri Mondor, who can be contacted at marjolaine.baude@aphp.fr.
